# Solubilization and renaturation of biologically active human bone morphogenetic protein-4 from inclusion bodies

**DOI:** 10.1016/j.btre.2018.e00249

**Published:** 2018-04-04

**Authors:** Gesa-Maria Gieseler, Kimia Ekramzadeh, Volker Nölle, Svitlana Malysheva, Henning Kempf, Sascha Beutel, Robert Zweigerdt, Ulrich Martin, Ursula Rinas, Thomas Scheper, Iliyana Pepelanova

**Affiliations:** a Institute of Technical Chemistry, Leibniz University of Hannover, Germany; b Miltenyi Biotec GmbH, Bergisch Gladbach, Germany; c Department of Cardiothoracic, Transplantation and Vascular Surgery, Medical University Hannover, Germany

**Keywords:** Recombinant human bone morphogenetic protein-4 (rhBMP-4), Inclusion bodies, Refolding, Cation-exchange membrane chromatography

## Abstract

Biologically active human bone morphogenetic protein-4 (hBMP-4) was successfully produced in a prokaryotic host. For this aim, hBMP-4 cDNA was cloned in *Escherichia coli* (*E. coli*) and the protein was produced in a non-active aggregated form. After washing and solubilization, *in vitro* refolding of the rhBMP-4 monomer was performed using rapid dilution. In this study, different refolding conditions were tested for the dimerization of rhBMP-4 by one-factor-at-a-time variation. The dimerization process was found to be sensitive to pH, protein concentration and the presence of aggregation suppressors. In contrast, redox conditions and ionic strength did not impact refolding as expected. The dimer was separated from the remaining monomer, aggregates and host cell contaminants in a single step using cation-exchange membrane chromatography. The rhBMP-4 dimer produced in *E. coli* was biologically active as demonstrated by its capability to induce trophoblast differentiation and primitive streak induction of human pluripotent stem cells (hPSCs).

## Highlights

• Biologically active rhBMP-4 was produced in a prokaryotic host as inclusion bodies.
• Different refolding recipes were tested for optimal dimerization yield.
• One-step purification of dimer with cation-exchange membrane chromatography.
• The product induces trophoblast differentiation in induced pluripotent stem cells.
• Comparison between commercial rhBMP-4 from cell culture and product from *E. coli*.

## 1 Introduction

Bone morphogenetic protein-4 (BMP-4) is a member of the BMP-family, a group of growth factors, initially discovered for their ability to initiate bone formation when transplanted to ectopic sites [1]. BMP-4 is part of the transforming growth factor (TGF-β) superfamily and is widely expressed from early embryogenesis through adulthood [2]. It plays a key role in mesenchyme formation and the development of multiple organs. Additionally, BMP-4 also acts as a differentiation factor to hematopoietic and nerve cells and promotes epidermal commitment [[3], [4], [5]]. Mature BMP-4 is a homodimeric glycoprotein with a cystine knot [6].

BMP-4 has been produced by recombinant technology, in order to study its signaling or to exploit potential therapeutic effects. Commercially available rhBMP-4 is usually derived from NS0 and HEK293 cell lines [7], though other hosts, such as Chinese hamster ovary cells (CHO) [8] have also been used for production. Drawbacks to production in mammalian cell lines usually include low yields and high costs.

This has fueled motivation of some research groups to attempt rhBMP-4 production in simple microorganisms. For example, Huang et al. reported the production of codon-optimized human recombinant BMP-4 (rhBMP-4) in *Pichia pastoris*. The protein was successfully secreted in the media, but exhibited hyperglycosylation. In addition, mainly the monomer was secreted, which confirmed problems in the protein folding of the mature domain [9].

Reports of the recombinant production of hBMP-4 in prokaryotic systems are rare. Due to its cystine knot structure, BMP-4 is produced in a non-active aggregated form in expression systems such as *Escherichia coli* (*E. coli*) [10,11]. In this case, rhBMP-4 must be solubilized and refolded *in vitro* to its native form. The refolding procedure must lead to correctly folded monomer and the successful dimerization of the protein with the formation of a cystine knot.

The final product of refolding is a mixture of the correctly-folded dimer, BMP-4 monomer, host cell proteins, as well as various aggregates. The selective isolation of the dimer and removal of monomer, aggregates and host cell impurities is required in order to obtain the isolated native product. Since BMP-4 bears a large structural and homological resemblance to BMP-2, we hypothesize that it will be possible to adapt well-described rhBMP-2 protocols [12,13] to rhBMP-4 solubilization and refolding.

Only few protocols for the expression and purification of recombinant BMP-4 from *E. coli* have been published so far. Bessa et al. and Klösch et al. overexpressed BMP-4 containing a His-tag in *E. coli*, which might require tag removal for potential clinical applications [10,11]. In both cases, no refolding was attempted and the protein was tested for activity directly after solubilization without comparison to rhBMP-4 derived from animal cell culture.

In our study, we aimed to produce rhBMP-4 in *E. coli* without tags by using a simple refolding protocol involving rapid dilution. Due to the sequence homology between BMP-2 and BMP-4, we could start with a well-described protocol for BMP-2, which was then adapted for the refolding of BMP-4, by the addition of aggregation suppressors and optimization of parameters by performing a one-factor-at-a-time-variation. After performing a one-step separation of the refolding solution, we were able to isolate biologically active homodimeric rhBMP-4.

## 2 Materials and methods

### 2.1 Organism and growth conditions

The gene coding for hBMP-4 was synthesized as a codon-optimized sequence for *E. coli* and cloned into the expression vector pET24a. The cloning procedure was verified by DNA sequencing. The expression vector pET24a-hBMP4 was then transformed into the *E. coli* strain BL21(DE3) (Merck Millipore, USA).

Production of rhBMP-4 in *E. coli* was carried out in a fed-batch cultivation. In brief, lysogeny broth (LB) medium (10 ml) was used for shake flask cultures. The starter culture contained 10 μl kanamycin (50 mg/ml) and was inoculated with 1 ml of a glycerol stock and cultivated for 5 h at 37 ° C with agitation at 150 rpm. Afterwards, defined medium [500 ml containing 3.3 g KH_2_PO_4_, 8.9 g Na_2_H_2_PO_4_*2H_2_O, 1.65 g (NH_4_)_2_SO_4_, 20 g/l glucose, 0.5 ml of kanamycin (50 mg/ml)] was inoculated with the starter culture and incubated at 37° C for 10 h at 150 rpm.

For the fed-batch process, the latter culture was transferred to a bioreactor (B. Braun, Germany) containing 7 l defined medium. Temperature was maintained at 37 ° C and pH at 7.0 by addition of 8 M NH_4_OH. The addition of glucose feeding solution was started after depletion of initial glucose as indicated by a dissolved oxygen spike. A growth rate of 0.2 h^−1^ was set (μ_set_) by using automatic feeding with a software controller based on a Monod model. After 4 h, the culture reached a relative OD_600_ = 50 and 1 mM isopropyl β-d-1-thiogalactopyranoside (IPTG) was added for induction of recombinant protein production. Cells were harvested by centrifugation at 5,500 × *g* for 25 min at 4 °C and stored at −80 °C. Approximately 65 g wet cell weight was obtained from 1 l culture. The frozen cell pellet was resuspended [20 g biomass in 100 ml 50 mM phosphate buffer of pH 7 at 4 °C] and the obtained slurry was pumped for 10 cycles at 7000 psi through a high pressure homogenizer (Microfluidics, USA). The obtained cell lysate was centrifuged at 5,500 x *g* for 15 min at 4 °C, followed by a washing step of the pellet with a buffer containing 20 mM Tris-HCl, pH 7.5, 0.5 mM ethylenediaminetetraacetic acid (EDTA) and 2% (v/v) Triton-X100. This step ensures removal of membrane proteins. The solution was centrifuged again at 26,000 × *g* for 60 min and the pellet containing the inclusion bodies was stored at −80 °C.

### 2.2 Solubilization of inclusion bodies and refolding of rhBMP-4

The purified inclusion bodies were incubated overnight at 4 °C in a solubilization buffer containing 20 mM Tris–HCl (pH 7.5), 150 mM NaCl, 5 mM dithiothreitol and 6 M urea. Remaining protein aggregates were separated by centrifugation at 26,000 × *g* for 15 min at 4 °C. The supernatant contained soluble BMP-4 at a concentration of 10 mg/ml.

The solubilized proteins were diluted 1:100 through rapid dilution into the refolding buffer consisting of 50 mM Tris-HCl (pH 8.5), 5 mM EDTA, 500 mM L-arginine and 33 mM 3-[(3-Cholamidopropyl)dimethylammonio]-1-propanesulfonate (CHAPS), 2 mM oxidized glutathione (glutathione disulfide, GSSG) and 1 mM reduced glutathione (GSH) and were incubated for 4 days. After refolding, the solution was centrifuged for 15 min at 4,000 × *g* to remove aggregated protein and was sterile-filtered (0.2 μm). This refolding buffer represents the standard recipe in this work, which was obtained by variation of components and testing for optimal yield. As a starting point, different concentrations of CHAPS (0–50 mM) and L-arginine were tested (0–800 mM). After determining the optimal concentration of CHAPS and L-arginine, the standard buffer recipe containing both additives was used as the basis for additional experiments. A variation of pH value, redox system and salt concentration was performed, whereas protein concentration (100 μg/ml) and incubation time (4 days) were maintained constant in all experiments.

### 2.3 Downstream processing of BMP-4 by cation-exchange membrane chromatography

The lab-scale purification of rhBMP-4 was performed using a low-pressure ÄktaPure (GE Healthcare, Sweden) chromatography system with an online UV absorbance detector at 280 nm and conductivity meters. Data was recorded using the accompanying software Unicorn (GE Healthcare, Sweden). All runs were operated at a flow rate of 1 ml/min.

Purification of rhBMP-4 was achieved by cation-exchange membrane chromatography (bed volume 3 ml, Sartobind^®^S nano module, Sartorius Stedim Biotech, Germany) at room temperature. Briefly, rhBMP-4 in the refolding solution was diluted 1:2 with a dilution buffer (20 mM Tris–HCl, 8 mM urea, pH 6) and was then directly applied to the membrane adsorber. Washing of the chromatographic membrane was performed with a buffer of 20 mM Tris–HCl, 4 M urea (pH 6). Finally, desorption of the bound proteins was performed with a step elution using a buffer of 20 mM Tris–HCl, 4 M urea and 1 M NaCl (pH 6). Removal of urea after membrane chromatography was achieved by buffer exchange with 20 mM Tris-HCl, pH 8.5 in a desalting column (Vivaspin^®^, molecular weight cut-off (MWCO) = 3000 Da; Sartorius Stedim Biotech, Germany).

### 2.4 Reducing and non‐reducing SDS‐PAGE

Protein samples were analyzed with sodium dodecyl sulfate-polyacrylamide gel electrophoresis (SDS‐PAGE) in a Mini Protean Tetra cell^®^ (Bio-Rad, Germany) using 4–20% Mini-PROTEAN^®^ TGX™ Precast Protein Gels, 15-well. Loading amount was 7 μl for chromatography fractions. Gels were stained with colloidal Coomassie Brilliant Blue solution. The purified BMP-4 dimer was analyzed with and without the reducing agent dithiothreitol (DTT) and boiled for 5 min at 95 °C before loading. Destained gels were scanned on an Epson perfection V750 pro scanner. For analyzing the extent of dimerization during the refolding process, the samples were treated with iodoacetamide (final concentration 0.1 mol/l). This alkylating reagent is commonly used for blocking thiols and prevents further disulfide exchange reactions. Protein concentration and purity was determined *via* gel densitometry calibration curve with pure rhBMP-4 as a standard (R&D Systems, USA). Gel densitometry was also used to calculate the refolding yield during dimer formation. Refolding yield is expressed as percentage of dimerized rhBMP-4 dimer (after 4 d of incubation, endpoint of renaturation) compared to total rhBMP-4 concentration (starting point of renaturation).

$$Refolding\;yield\left[\%\right]=\frac{rhBMP-4\;dimer\;\left(end\;point\;of\;refolding\right)\left[\mu g\right]}{rhBMP-4\;monomer\left(starting\;point\;of\;refolding\right)\left[\mu g\right]}$$

### 2.5 Endotoxin assay

The endotoxin content of the purified BMP-4 was determined by the *Limulus amebocyte* lysate (LAL) assay. The test was carried out using an Endosafe^®^-PTS™ system (Charles River Laboratories, USA) with a cartridge ranging from 0.1–10 EU ml^−1^ (EU = endotoxin unit) and following the manufacturer’s instructions.

### 2.6 BMP-4 activity assay by primitive streak induction

The capacity of BMP-4 to quantitatively promote primitive streak induction in human embryonic stem cells (hESC) was determined by using a MIXL1-GFP reporter cell line [14], as described previously [15]. In brief, single-cell suspensions were seeded in 12-well suspension plates (Greiner, Austria) at 0.33 × 10^5^ cells/ml in mTeSR medium (Stem Cell Technologies, Canada) supplemented with 10 μM Y-27632 inhibitor (kindly provided by Gerald Dräger, Leibniz University Hannover, Germany). Suspension aggregates formed within 24 h h. After 4 days, primitive streak induction was initiated by changing the medium to RPMI medium supplemented with 2% B27 supplement (Life Technologies, USA), 3 μM CHIR99021 inhibitor (kindly provided by Gerald Dräger, Leibniz University Hannover, Germany) and BMP-4 concentrations as indicated in the results section. Commercial rhBMP-4 (R&D Systems, USA) from NS0 mammalian cell culture was used as a positive control. Aggregates were harvested after 24 h and dissociated using Accutase for 5 min at 37 °C. The GFP signal was measured with a BD Accuri C6 flow cytometer (BD Biosciences, USA) and analyzed using FlowJo (v10; TreeStar, FlowJo LLC, USA).

### 2.7 BMP-4 activity assay by induction of trophoblast differentiation

BMP-4 is known to trigger the differentiation of human induced pluripotent stem cells (hiPSCs) into trophoblast-like cells [16]. HiPSCs were cultured in our own in-house produced Essential 8 medium (E8). The trophoblast differentiation protocol was modified from Amita [17]. Briefly, cells were plated at low density (4 × 10^3^ cells per cm^2^) in Matrigel-coated cell culture plates in E8 medium. After 48 h, the E8 medium was replaced with differentiation medium containing 1 μM A8301 (Tocris, UK) and 0.1 μM PD173074 (Sigma, US), as well as 10 ng/ml commercial BMP-4 (R&D Systems, USA) or the rhBMP-4 dimer produced in *E. coli*. In 48 h after the addition of the differentiation medium, the cells were fixed with 4% paraformaldehyde and immunostained for cdx2, a transcription factor known to be an early trophoblast marker. The primary antibody was an Anti-CDX2, clone CDX2-88 (BioGenex, US) and the secondary antibody was Alexa-Fluor-488 conjugated donkey-anti-mouse (Jackson ImmunoResearch, US). Counterstaining was performed with 4′,6-diamidino-2-phenylindole (DAPI).

## 3 Results and discussion

### 3.1 Production, solubilization of inclusion bodies and *in vitro* refolding of rhBMP-4

**Fig. 1:**
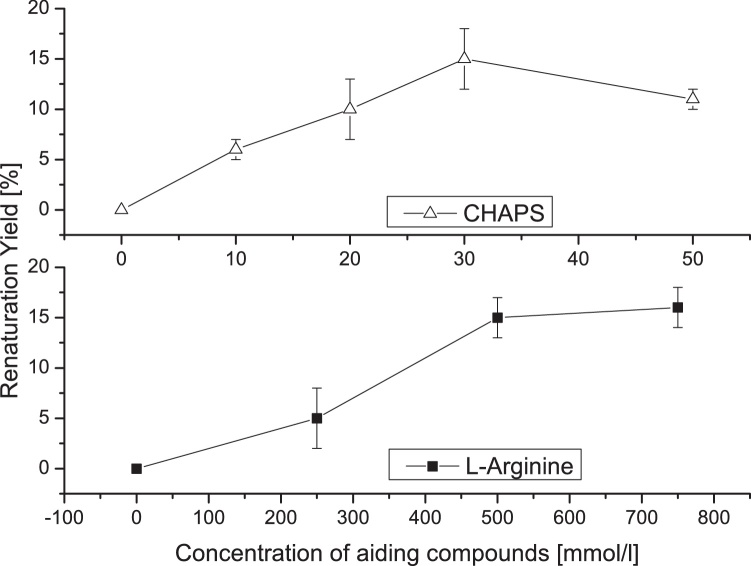
Effect of different aiding compounds on the in-vitro dimerization of rhBMP-4. Additives were adjusted to the indicated concentrations.

A fed-batch strategy was used to achieve high cell concentrations and thereby improve productivity of recombinant protein production. In this manner, rhBMP-4 was overexpressed in inclusion bodies. These were solubilized as described in the “material and methods” section and subjected to *in vitro* refolding. Initial refolding attempts based on BMP-2 renaturation protocols [12,13] resulted in aggregation of the protein and did not lead to dimer formation. Alternative additives were included in the renaturation protocol which helped to solubilize the proteins and prevent precipitation. Commonly used additives for promoting protein refolding include amino acids (e. g. L-arginine) and detergents such as CHAPS. Rapid dilution of solubilized inclusion bodies was performed into buffers containing different concentrations of L-arginine and CHAPS. Increasing the amount of L-arginine and CHAPS enhanced the refolding yield of rhBMP-4. The combination of both additives at their optimal concentrations did not result in refolding yields significantly exceeding 15% (see Fig. 1).

Renaturation was performed using protein concentrations between 50 and 100 μg/ml. Higher amounts of protein resulted in protein aggregation, thereby drastically reducing the refolding yield of soluble protein.

### 3.2 Effect of pH, redox-conditions and salt concentration on refolding of rhBMP-4

**Fig. 2:**
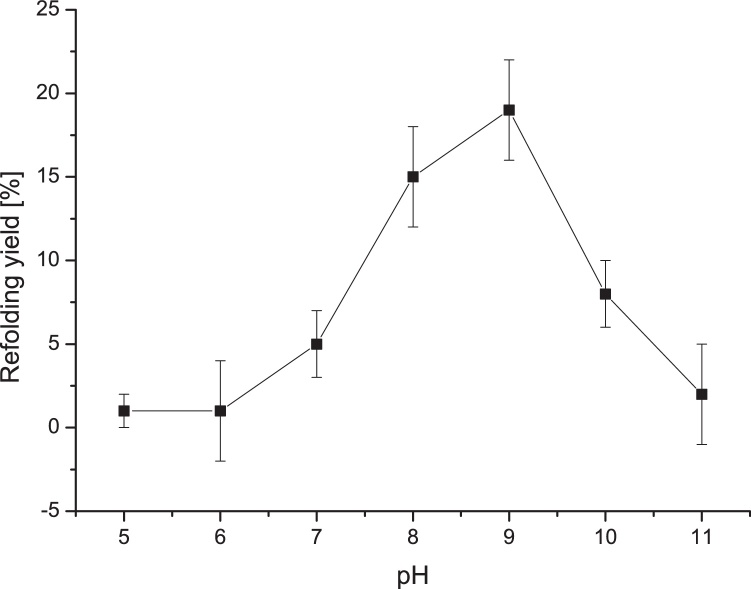
Effect of pH on the refolding yield of rhBMP-4. Here, the pH was adjusted to the indicated values. The standard composition of refolding buffer was used for all other parameters.

Folding and protein association leading to aggregated proteins are competing processes which strongly depend on the pH and redox system. Disulfide bond formation and reshuffling reactions are accelerated at the highest pH where the protein can still maintain its native structure [18]. To find optimal conditions for BMP-4 refolding, different pH values were tested for renaturation (see Fig. 2). In this experiment, low renaturation yields were determined at pH-values <8. This is probably related to the fact that pH values below the pK_a_ value of the reactive thiolate anion (pK_a_ = 8.5) are not supporting disulfide bonding and reshuffling. At higher pH (pH > 9) renaturation yields diminished as well. This relatively narrow pH window is not unusual for proteins containing disulfide bonds [19]. Here, rhBMP-4 shows similarities to rhBMP-2, in that it exhibits only a narrow range for renaturation compared to other proteins containing cystine knots [19,20].

**Fig. 3:**
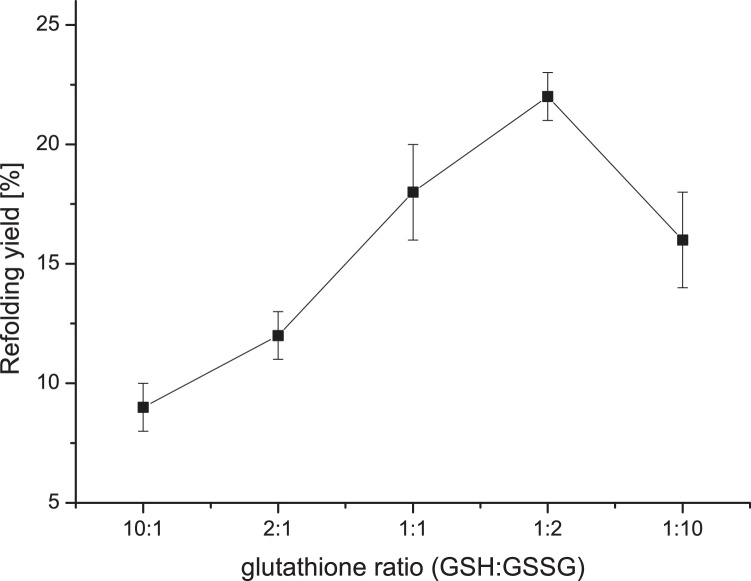
Effect of different redox systems on the refolding yield of rhBMP-4. Here, glutathione concentrations were set to the indicated ratios of reduced to oxidized glutathione form (GSH:GSSG). Standard composition of refolding buffer was used for all other parameters.

Mixtures of reduced and oxidized glutathione are frequently used for formation of the cystine knot. Usually, an excess of the reduced thiol component facilitates thiol exchange and leads to higher renaturation yields. Fig. 3 illustrates how a variation in redox conditions impacted the refolding yield of rhBMP-4. The optimal conditions required two-fold excess of the oxidized glutathione but the overall yield did not improve significantly.

**Fig. 4:**
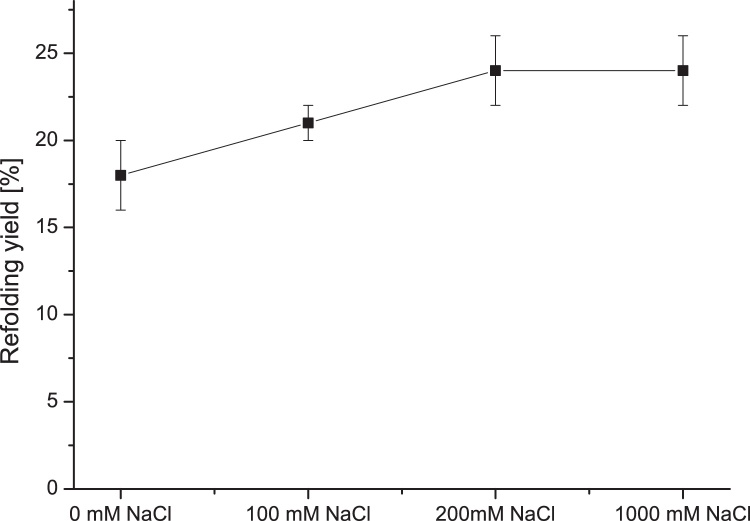
Effect of different concentrations of NaCl on the refolding yield of rhBMP-4. NaCl concentration was adjusted to the indicated values. Standard composition of refolding buffer was used for all other parameters.

Similar trends were determined using sodium chloride at different concentrations during the refolding process. In general, at low concentrations, sodium chloride inhibits electrostatic interactions, thereby reducing protein aggregation [21]. Concentrations of salt sometimes in the range of up to 1000 mM have been reported to stabilize folding and reduce aggregation [22,23]. At some point, however, high salt concentrations will cause the opposite effect, leading to exposure of hydrophobic residues in the protein core and increased aggregation. Fig. 4 shows enhanced refolding yields with increasing sodium chloride concentrations. In this case as well, the overall refolding yields did not exceed 24% which indicates that sodium chloride content has a minor impact on the refolding yield of rhBMP-4. Additionally, salt concentrations above 200 mM up to 1000 mM show no further improvement on refolding yield at all.

**Fig. 5:**
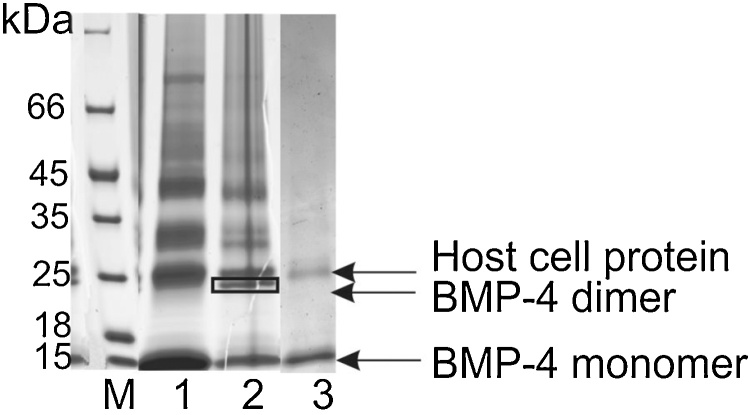
Coomassie-stained, non-reduced SDS-PAGE showing the refolding process to rhBMP-4 dimer. 1: Sample of renaturation process at t_0_, 2: Sample of renaturation after 4 days incubation time (end point) showing BMP-4 dimer in the marked square and a host cell protein band above, 3: End point of refolding applied on a reducing gel, M: protein ladder #26610 (Thermo Scientific).

Dimerization was completed after an incubation time of 4 days, using the optimized composition of refolding buffer (as described in the standard recipe in the “material and methods” section). Fig. 5 illustrates the formation of the dimer resulting in a new band at 26 kDa. At the indicated position in lane 2, two distinct protein bands can be observed at a similar height. The upper band is a host cell protein and the lower one is BMP-4 as shown by applying a sample from the endpoint of refolding (4 days) on a reducing gel shown in lane 3. Here, the BMP-4 dimer band is reduced to its monomeric form, while the host cell protein band remains intact. The end of the refolding process results in a mixture of monomer, dimer and structures with higher molecular weight, which necessitates a separation process for the dimer form.

### 3.3 Downstream processing of BMP-4

**Fig. 6:**
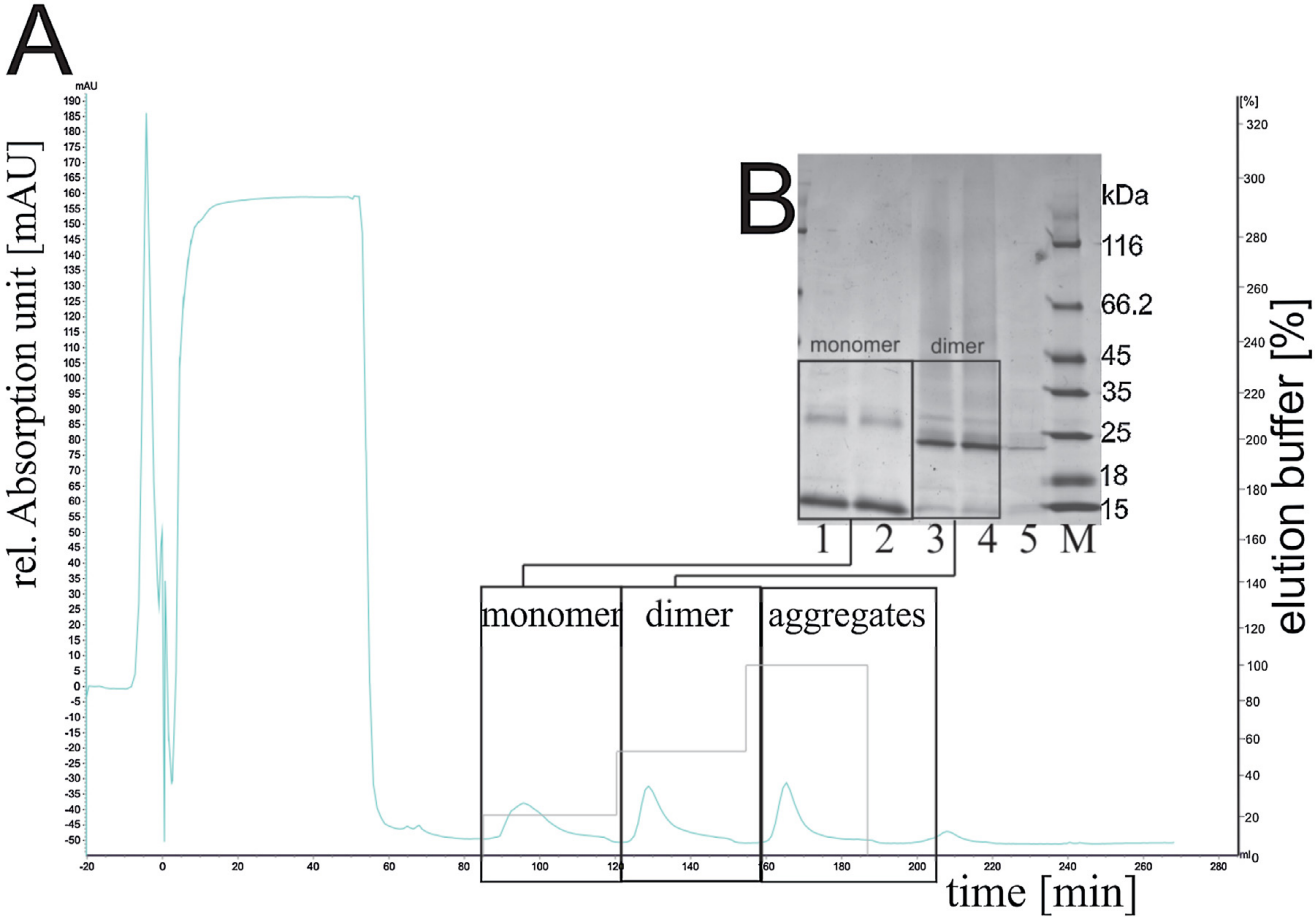
a: Chromatogram of rhBMP-4 purification using cation-exchange membrane chromatography. Three steps were used for the elution of bound proteins (containing 150 mM NaCl, 300 mM NaCl and 500 mM NaCl). UV absorbance at 280 nm is shown. Flow rate was set to 1 ml/min. b: Coomassie-stained non-reduced SDS-PAGE: 1&2: elution fraction monomer, 3&4: elution fraction dimer, 5: aggregates, M: protein ladder #26610 (Thermo Scientific).

After solubilization and renaturation, rhBMP-4 was applied to a cation-exchange membrane adsorber (Sartobind^®^ S, Sartorius Stedim Biotech, Germany) for the purpose of separation of the dimer from other components of the refolding mixture. The majority of host cell proteins were eluted with the washing step (data not shown). Desorption was performed with three consecutive elution steps (see Fig. 6). Monomeric rhBMP-4 eluted with the first isocratic step (100 mM NaCl), while desorption of the dimer occurred at 300 mM NaCl during the second isocratic step. The isolated BMP-4 dimer appeared as a single major protein band with > 80% purity on non-reduced SDS–PAGE. Aggregates and traces of BMP-4 dimer eluted within the third elution step (500 mM NaCl). This order of elution is to be expected in ion-exchange chromatography. The BMP-4 dimer shows stronger affinity for the chromatographic phase compared to the monomeric form alone. Aggregates and higher structures bind even more strongly to the chromatographic membrane, which allows the selective isolation of the dimer under appropriate conditions of ionic strength. After cation-exchange membrane chromatography, the identity of resulting fractions was confirmed using mass spectrometry (data not shown). The isolated BMP-4 dimer was dialyzed and tested for biological activity in cell culture experiments. The final yield obtained in the process after purification and dialysis was 75.6 mg rhBMP-4 dimer per liter of culture broth.

### 3.4 Endotoxin content and assays of biological activity

The endotoxin level is a critical parameter for cytokines used in cell culture. It was determined by the LAL assay. The measured value of 95 EU/mg fits requirements for low endotoxin levels (<1 EU/μg protein) for commercial cytokines produced in recombinant *E. coli.*

**Fig. 7:**
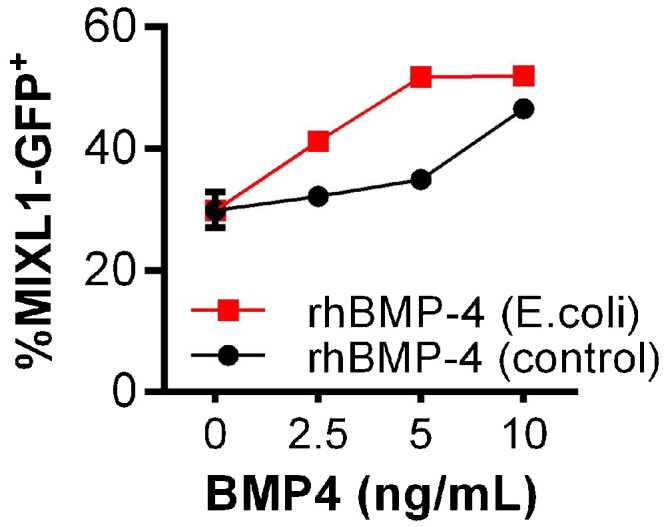
Flow cytometric analysis of MIXL1-GFP expression, a gene expressed during primitive streak formation, after 24 h stimulation at different concentrations of rhBMP-4 produced in *E. coli* and commercial rhBMP-4 (positive control).

The biological activity of the purified rhBMP-4 was tested and compared to commercial rhBMP-4 from mammalian cell culture in two different biological assays, both based on human pluripotent stem cells. The first assay tested the dose-response of rhBMP-4 on primitive streak induction using a MIXL1-GFP reporter cell line [14]. This embryonic stem cell line carries a green fluorescent protein (GFP) in the endogenous MIXL1 locus, an established marker for primitive streak formation. The induction of the primitive streak is prerequisite for the formation of mesendodermal derivatives in the human body and is strongly dependent on WNT, BMP and TGF-signaling [24,25]. The rhBMP-4 produced in this study promoted the primitive streak induction in a typical dose-dependent fashion (Fig. 7). Moreover, its activity was comparable to the positive control. The background expression (ca. 30%) without BMP-4 addition is caused by the action of the CHIR99021 molecule, a WNT pathway activator.

**Fig. 8:**
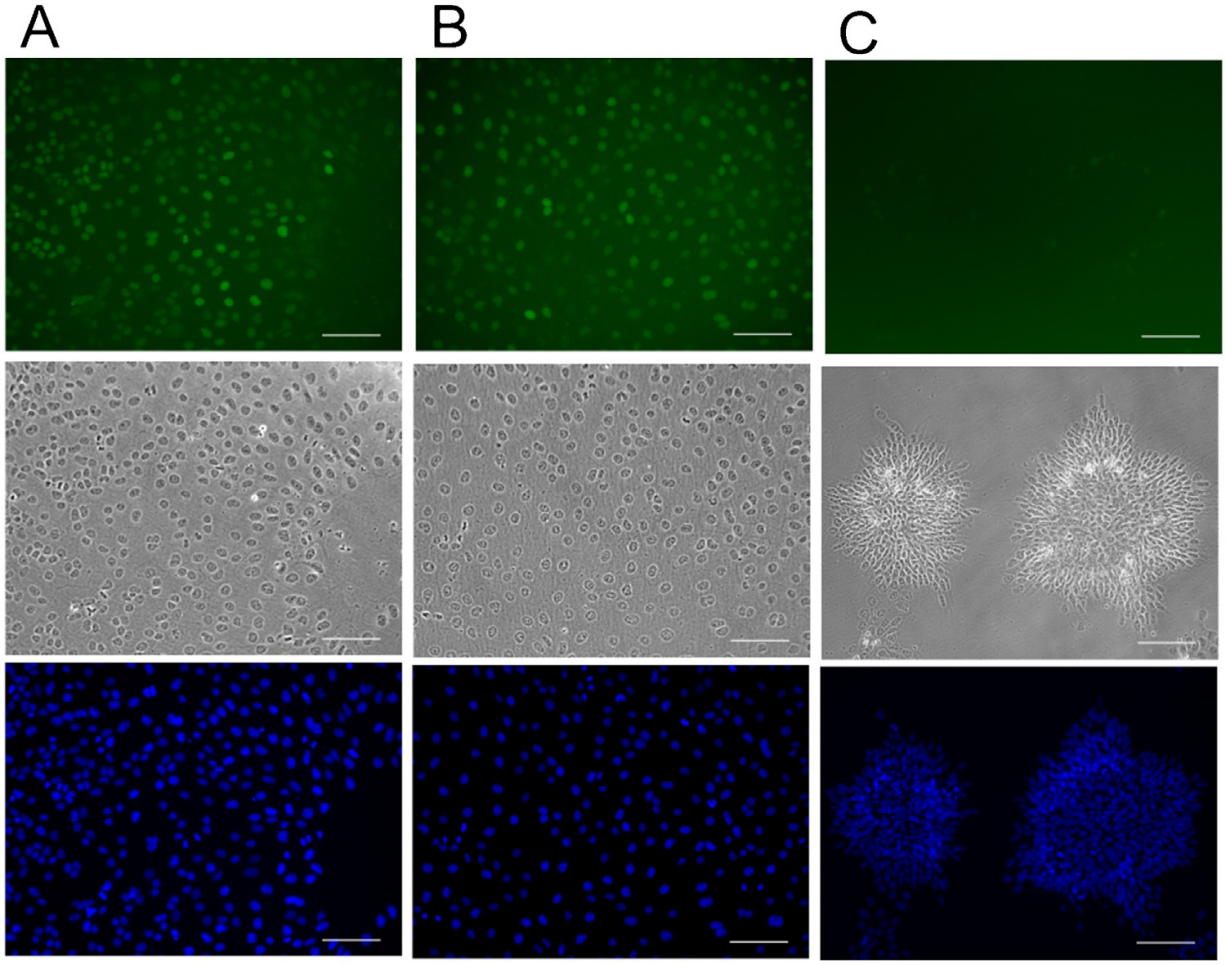
HiPSCs after 48 h in differentiation medium with A: *E. coli*-produced rhBMP-4 (conc. 50 ng/ml), B: commercial rhBMP-4 (conc. 10 ng/ml) or C: without BMP-4 addition (negative control). Cdx-2 positive cells were stained green (upper row), cells with rhBMP-4 demonstrated typical cobblestone morphology on the second day of differentiation (middle row), cell nuclear counter staining with DAPI (last row), scale-bar 100 μm.

The second assay tested the effect of the purified rhBMP-4 on the trophoblast differentiation of human induced pluripotent stem cells (hiPSCs). Here, cdx2 is identified as an early trophoblast marker and cells were stained for its presence. On the second differentiation day, cells with the control (rhBMP-4 from R&D Systems, 10 ng/ml) and with the rhBMP-4 dimer produced in this study (50 ng/ml), demonstrated typical cobblestone-like trophoblast morphology and expressed cdx2 in the nuclei. The rhBMP-4 dimer from *E. coli* did not display biological activity at a concentration of 10 ng/ml (data not shown). Without the addition of BMP-4 (negative control), the cells failed to form a cobblestone-like monolayer and did not express cdx2 (Fig. 8).

## 4 Conclusion

In this contribution, we present a new strategy suitable for the production of recombinant human BMP-4 in *E. coli*. The protein was produced in the form of inclusion bodies and had to be solubilized and refolded by finding suitable renaturation buffer conditions. Mild solubilization without refolding wasn’t attempted, because studies suggest that cystine knot proteins cannot be obtained in an active form in this manner [26]. Therefore, different compositions of refolding buffers were tested for the achievement of optimal renaturation yield. The final product of refolding included a mixture of dimer, monomer and higher aggregates. Thus, purification was necessary to isolate the dimer from the refolding mixture selectively, in order to test for biological activity. Separation of the refolding mixture was performed by a single purification step involving cation-exchange membrane chromatography with step elution. A quantitative assay of rhBMP-4 activity showed a dose-dependent promotion of primitive streak induction and confirmed that the produced dimer is correctly folded and biologically active. The biological activity of the *E. coli*-derived rhBMP-4 was also examined by its ability to induce the transcription factor cdx-2, an early marker of trophoblast differentiation. This analysis also indicated that rhBMP-4 from *E. coli* is biologically active; however, the determined activity here was lower than NS0 cell culture-derived rhBMP-4. The reason for reduced bioactivity in the bacterial rhBMP-4 could very well be the lack of glycosylation patterns, as previously shown *in vitro* for rhBMP-2 produced in prokaryotic systems [27]. In addition, the smears visible in the gel after purification in Fig. 6b indicate that BMP-4 aggregates are still present in the sample. These surely diminish the overall bioactivity of the isolated product.

Nevertheless, the process for rhBMP-4 production presented here demonstrates that it is feasible to produce biologically active rhBMP-4 dimer from *E. coli*. The process is marked by simplicity and can be easily up-scaled through the use of a larger membrane module.

## Conflict of interest

The authors declare no conflict of interest.
